# Current status of diagnosis and treatment of primary benign cardiac tumors in children

**DOI:** 10.3389/fcvm.2022.947716

**Published:** 2022-10-21

**Authors:** Chuqiao Sheng, Chunfeng Yang, Yongkang Cheng, Yu-Mei Li

**Affiliations:** Department of Pediatric Intensive Care Unit, The First Hospital of Jilin University, Changchun, China

**Keywords:** primary cardiac tumors, cardiovascular imaging (CV imaging), cardiovascular pathology, children, benign cardiac tumors

## Abstract

Primary cardiac tumors in children are exceedingly rare overall, which benign account for most part. The onset of the disease is occult, while the clinical manifestations are non-specific-patients may be asymptomatic or show a range of obstructive, arrhythmic, embolic or systemic symptoms. The clinical presentations generally depend on the tumors’ size, localization, and pace of growth of the tumor. Moreover, the diagnosis needs comprehensive judgment based on imaging results and pathological examination. With advances in cardiac imagining and the introduction of cardiopulmonary support, the diagnosis and treatment of these rare tumors have improved the prognosis and outlook for benign tumors. To sum up the above, we sought to integrate articles from recent years for the latest comprehensive review of the clinical manifestations, imaging characteristics, clinic pathologic features and treatment of benign cardiac tumors in children to provide a broader idea for pediatricians to recognize and treat such diseases.

## Highlights

– In this review, we systematically summarize the clinical characteristics and auxiliary examination characteristics of benign cardiac tumors, which is particularly important for improving the clinical thinking of pediatric physicians
– Previously published literature on cardiac tumors has focused more on summarizing treatment approaches and survival times. We summarize the latest technical characteristics
– The critical point of this review is to improve the systematic understanding of pediatricians and medical technicians on benign cardiac tumors.

## Key message

The clinician’s ability to recognize and diagnose the disease is critical in diagnosing and treatment of primary cardiac tumors in children. Using non-invasive examination and accurate judgment can reduce the misdiagnosis rate of children; reduce the psychological burden of invasive examination and family. Therefore, developing compassionate diagnosis and treatment methods is more important than treatment methods.

## Background

Primary cardiac tumors are exceedingly rare in all age groups, with an approximate incidence of 0.0017–0.17% in autopsy series or during the clinical examination (1–3), it is difficult to obtain accurate statistics because of the unbalanced means of medical examination globally. Due to the development of non-invasive imaging modalities in recent years, the reports of primary cardiac tumors have gradually increased. Among these diagnosed cardiac tumors, benign tumors account for approximately 90% (3, 4). In a retrospective analysis by Morka et al. (1) in Poland, 95.1% of children diagnosed with a heart tumor were determined to be benign primary cardiac tumors in multiple centers over 15 years. Coincidentally, in another retrospective analysis by Wang et al. (5) it was mentioned that among the 52 children with primary cardiac tumors treated in the past 30 years in China, 48 (92.3%) cases were benign tumors. However, benign tumors can adversely affect children’s health, such as syncope, seizures, heart failure, arrhythmia and sudden death (5, 6). With the advancement of clinical diagnostic technology, the detection rate of children’s cardiac tumors has increased, and clinicians’ awareness of primary cardiac tumors has gradually improved. Based on the improvement of diagnostic technology for primary cardiac tumors in recent years. This systematic review aims to improve the thinking of pediatricians in the systematic diagnosis and treatment of children with primary benign cardiac tumors.

## Nomenclature and classification

The current nomenclature and classification of cardiac tumors mainly adopt the World Health Organization (WHO) classification of cardiac tumors (Fourth Edition) (7). Unlike the incidence in adults, the most frequent tumors reported in children were rhabdomyoma, followed by fibroma, myxoma, teratoma, and hemangioma (1, 4, 8). When analyzing the age-adjusted prevalence of tumors, rhabdomyoma, hemangioma, and fibroma were more common among infant, while myxoma was more common among children over 1 year old (8).

## Clinical manifestation

Children with cardiac tumors can be consists of non-specific symptoms-just like fever, and anemia, which make the diagnosis challenging, or present with a variety of clinical traits depending on the tumors’ size, localization, and pace of growth (9, 10). The clinical presentation generally does not depend on the histopathology of the tumor, so there was no significant difference in mass effect between benign and malignant cardiac tumors. Tumors can occur in the myocardium, atrium (chamber) cavity, and heart valve. When the tumor is small, there may be no apparent symptoms; when the tumor is massive, the obstruction is severe and narrowing of the cardiac chamber. The extensive tumor infiltration into the ventricular wall will decrease the effective myocardial beat, resulting in weakened cardiac systolic and diastolic functions. Arrhythmias may occur when tumors involve the conduction system, including premature beats, supraventricular tachycardia, pre-excitation syndrome, bundle branch block, etc.; Among these, atrioventricular block and ventricular tachycardia are not uncommon and may even be related to sudden death. When the tumor is located around the biventricular inflow (outlet) tract, it may cause obstruction, and the blood flow velocity increases at the location of the obstruction, resulting in a heart murmur. Atrial mass is more likely to block the flow of atrioventricular blood flow than ventricular mass, similar to valve stenosis, and ventricular mass blocking the outflow tract may lead to chest pain, dyspnea, and syncope. In addition, tumor infiltration of surrounding organs or tissues may cause the corresponding dysfunction. It should be emphasized that cardiac myxomas that easily dislodge fragments in older patients may cause multi-organ embolism (11–13). The possible clinical manifestations of each tumor will be discussed in detail.

## Diagnostic approach

All examination methods are widely used in adult patients or older children. At present, the diagnosis of cardiac tumors still mainly relies on echocardiography. Most clinical reports mentioned that echocardiography was complemented with magnetic resonance imaging, computed tomography, and histopathological examination for diagnosing cardiac tumors. Although percutaneous or catheter biopsy can directly determine the histological type, its was not suitable for pediatric patients due to its shortcomings, such as invasiveness and poor repeatability (Table 1). It is recommended to explore new methods suitable for children-specific examinations in the process of diagnosis and operation. A complete and consistent examination and high-quality imaging are essential for improving diagnostic accuracy. The tumor type and mass effect should be clarified for children with suspected cardiac tumors to guide the follow-up treatment.

**TABLE 1:** Outline advantages/disadvantages, indications, and main findings of different investigative modalities.

Diagnostic approach	Appliance	Advantage	Disadvantage	Main findings
Echocardiography	Can make a comprehensive, direct, and sensitive assessment of structural imaging, blood flow rate, and cardiac function	Non-invasive and pivotal in assessing blood flow and the degree of obstruction	Echocardiography offers relatively poor visualization of soft tissue and cardiac tumor infiltration compared to CT and MR imaging	(i) The heart is enlarged, and there may be moderate to large pericardial effusion; (ii) the tumor can be nodular, located in the submucosal or muscular layers of the atrium and ventricle, and can protrude into the pericardial cavity. The growth location, inside echo, and activity of the tumors, are typically different; (iii) the local blood flow velocity changes significantly if the tumor affects the outflow (inlet) tract.
Two-dimensional transthoracic echocardiography (TTE)		Describe the relationship between tumor location, size, number, shape, activity, and surrounding tissue structure	It lacks the advantage of displaying low-velocity blood flow	
Doppler ultrasound		Evaluate the hemodynamic changes caused by the mass effect of the tumor	Excessive thoracic gas affects the examination	
Transesophageal echocardiography (TEE)		(i) More apparent in diagnosing intracardiac mass lesions than conventional two-dimensional ultrasound; (ii) provide further details and higher accuracy in case a deeper examination; (iii) clearly show small thrombus	Limited by the skill level of the operators and the degree of cooperation of the child	
Contrast-enhanced myocardial ultrasonography (CEUS)		Qualitative, semi-quantitative, and quantitative methods to observe the blood flow inside the cardiac mass with high safety, which is helpful for qualitative and quantitative diagnosis of cardiac tumors		
Cardiac MRI (C-MRI)	It reflects the tumor’s nature and positional relationship with surrounding tissues before surgery.	C-MRI has a larger field of view and multi-plane three-dimensional imaging capabilities and is better than echocardiography in reflecting tumor properties and the positional relationship with surrounding tissues.	(i) The patient should fast for 4-6 h before the examination; (ii) general anesthesia or sedation is often required during cardiac MRI examinations in infants and young children, and cardiac MRI may be biased in diagnosing children with a basal heart rate too fast; (iii) clinical popularity is not as good as that of cardiac ultrasound.	The main imaging signs are (a)—infiltration or compression of the heart or large blood vessels by tumor cells; (b). The epicardium is separated from the endocardium, showing a low-density area between them; (c). The soft tissue mass locates in the cardiac cavity.
Chest computed tomography (CCT)	CCT might be preferred for patients with suspected malignant tumors and possible metastases	(i) Show the adjoining relationship between tumor and mediastinum, great blood vessels, extracardiac tissue involvement, and tumor activity; (ii) according to the CT value, the blood supply and histological characteristics (calcification, adipose tissue, etc.) of the tumor; (iii) can provide evidence for determining surgical indications and surgical options	CCT is associated with exposure to ionizing radiation	It can sensitively display the location of cardiac tumor involvement, the degree of invasion of the myocardial wall, the size of the tumor, the number of cardiac tumors, and different degrees of pericardial effusion, etc.
Cardiac enhancement CT	Identify cardiac tumors and thrombus	This examination can be considered for abnormal coagulation function in	A high-dose contrast agent must be injected during CT-enhanced	
		children	scanning, which is unsuitable for patients with critical illness and a contrast agent allergy	
Cardiac CT Angiography (CCTA)	CCTA may be considered in children with abnormal coagulation function	(i) Relatively non-invasive; (ii) it has high spatial resolution and three-dimensional assessment capabilities, which can provide information on tumor vascularity and possible involvement of coronary arteries and determine whether tumors have abnormal vascular branches;	Appropriate sedative drugs need to be given during the examination	
Fluorodeoxyglucose/PET (FDG-PET)	A complementary tool for evaluating patients with cardiac mass and can help differentiate benign from malignant lesions	Identify whether it is a secondary metastases and determine the thickness of the lesions	Large amount of radiation exposure, short inspection time	Clearly display the location, number, and richness of tumor blood flow in the heart
Plain chest X-ray	Determine whether the cardiac shadow is enlarged and pleural effusion	Non-invasive, small radiation, clear intrathoracic coronal and sagittal structures	It cannot show the space-occupying space in the cardiac cavity or the space-occupying volume in the myocardial wall, and it is not specific for tumor diagnosis	Enlarged heart with non-specific
Electrocardiogram (ECG)	ECG can show different types of arrhythmias if the tumor involves the conduction system of the heart in different locations	Non-invasive, repeatable operation, monitoring heart rhythm and heart rate at any time	No specific changes for tumors that do not involve the cardiac conduction system	If the tumor affects the cardiac conduction system, atrial or ventricular premature beats and conduction block may occur, and in severe cases, ventricular tachycardia may occur.
Cardiac catheterization	An adjunct diagnostic method that can be used as appropriate	One of the means of minimally invasive examination and treatment with minimal trauma, quick recovery	It requires invasive operations, may induce complications such as infection and thrombosis and has poor repeatability, which is difficult for infants and young children to tolerate	-
Pathological biopsy	For all tumors removed by surgical excision (Needle Puncture)	Gold standard	(i) This approach is limited to patients undergoing surgical resection; (ii) there are many differences between the pathological morphology of primary cardiac tumors in the pediatric population and primary cardiac tumors in adults.	Different tumors have different appearances under the microscope and have unique manifestations
Serological examination	One of the auxiliary diagnostic methods	Continuous monitoring to assist in assessing cardiac function, physiological conditions, etc.	Changes were not specific	There may be elevated white blood cell counts, blood platelet counts or proinflammatory cytokines, with no specific.

### Echocardiography

**FIGURE 1:**
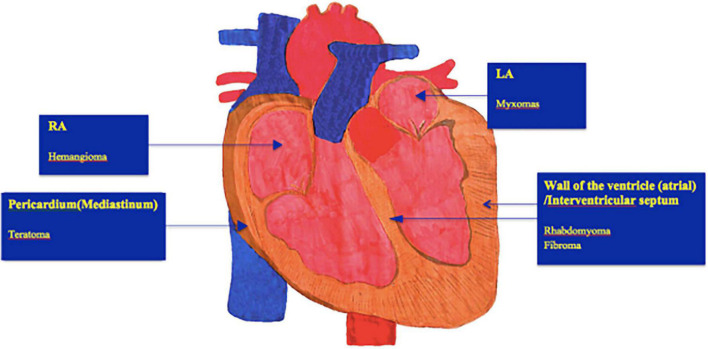
Show typical locations of different subtypes of benign cardiac tumors. However, many heart tumors can occur in any chamber. LA, Left atrium; RA, Right atrium.

Workup of a suspected cardiac tumor should begin with echocardiography since it is non-invasive and pivotal in assessing blood flow and the degree of obstruction. Echocardiography has high value for diagnosing cardiac tumors-can make a comprehensive, direct, and sensitive assessment of structural imaging, blood flow rate, and cardiac function, where it is the most frequent imaging modality employed (14), so its application runs through the entire treatment and follow-up process of children with cardiac tumors. The main imaging findings under ultrasound: (i) The heart is enlarged, and there may be moderate to large pericardial effusion; (ii) the tumor can be nodular, located in the submucosal or muscular layers of the atrium and ventricle, and can protrude into the pericardial cavity. The growth location, inside echo, and activity of the tumors, are typically different (Figure 1); (iii) the local blood flow velocity changes significantly if the tumor affects the outflow (inlet) tract.

Two-dimensional transthoracic echocardiography (TTE) can accurately describe the relationship between tumor location, size, number, shape, activity and surrounding tissue structure, but it lack the advantage of displaying low-velocity blood flow. If the tumor is located in the pericardium, once pericardial effusion occurs, the tumor needs to be differentiated from the extrusion of extra-pericardial lesions (15). Doppler ultrasound can evaluate the hemodynamic changes caused by the mass effect of the tumor; transesophageal echocardiography (TEE) is more apparent in diagnosing intracardiac mass lesions than conventional two-dimensional ultrasound (16). TEE can provide further details and higher accuracy in case a deeper examination of the mass is needed. Moreover, TEE can also clearly show small thrombus that TTE cannot. However, its application in the pediatric patient group is limited by the skill level of the operators (17) and the degree of cooperation of the child; In addition, echo contrast agents are helpful to confirm the presence of an intracardiac mass and to characterize it further under the extent of contrast enhancement. There are qualitative, semi-quantitative, and quantitative methods to observe the blood flow inside the cardiac mass by contrast-enhanced myocardial ultrasonography (CEUS), with high safety (16, 18), which is helpful for qualitative and quantitative diagnosis of cardiac tumors. It has a high application value in diagnosing cardiac tumors (19). It is emphasized that echocardiography offers relatively poor visualization of soft tissue and cardiac tumor infiltration compared to CT and MR imaging. So, if it is difficult to determine the nature of the mass under ultrasonic cardiogram (UCG), further cardiac magnetic resonance imaging (MRI) or cardiac contrast-enhanced CT can be considered for supplementary diagnosis. Therefore, CT and MRI are increasingly important in diagnosing, characterizing, and planning treatment strategies for cardiac tumors.

### Cardiac magnetic resonance imaging

MRI is the most sensitive imaging technique. With the continuous development of new MRI technologies, various scanning sequences have been gradually applied in clinical practice. These technologies include 2D and 3D SSFP sequences and ECG-gated spin-echo T1W SEEPI sequences, significantly improving the time of MRI images and spatial resolution, multi-directional display of cardiac tumor morphology and its impact on cardiac function, which is of great significance for the clinical diagnosis of cardiac tumors. C-MRI has a larger field of view and multi-plane three-dimensional imaging capabilities and is better than echocardiography in reflecting tumor properties and the positional relationship with surrounding tissues (20, 21). The main imaging signs are: (a) Infiltration or compression of the heart or large blood vessels by tumor cells. (b) The epicardium is separated from the endocardium, showing a low-density area between them. (c) The soft tissue mass locate in the cardiac cavity.

A multicenter retrospective study by Beroukhim et al. (22) concluded that cardiac MRI has a 97% accuracy in predicting tumor histology, which is critical in establishing the best-individualized treatment plan and determining the need for surgical intervention. Thence, tissue characterization of the tumor by cardiac MRI is strongly recommended before cardiac surgery (23). However, the patient should fast for 4–6 h before the examination, general anesthesia or sedation is often required during cardiac MRI examinations in infants and young children, and cardiac MRI may be biased in diagnosing children with a basal fast heart rate. Clinical popularity is not as good as that of cardiac ultrasound.

### Chest computed tomography

Chest CT can see the space-occupying lesions in the heart, which has a definite diagnosis and differential diagnosis significance and can show the adjoining relationship between tumor and mediastinum, great blood vessels, extracardiac tissue involvement, and tumor activity. According to the CT value, the blood supply and histological characteristics (calcification, adipose tissue, etc.) of the tumor can also be inferred to provide better guidance for clinical treatment. Whereas CT is associated with exposure to ionizing radiation, it might be preferred for patients with suspected malignant tumors and possible metastases. This examination can provide evidence for determining surgical indications and surgical options.

Cardiac enhancement CT is mainly used to identify cardiac tumors and thrombus. This examination can be considered for abnormal coagulation function in children (24). However, a high-dose contrast agent must be injected during CT-enhanced scanning, which is unsuitable for patients with critical illness and a contrast agent allergy (25). Cardiac CT Angiography (CCTA) is relatively non-invasive. It has high spatial resolution and three-dimensional assessment capabilities, which can provide information on tumor vascularity and possible involvement of coronary arteries and determine whether tumors have abnormal vascular branches. During this examination, appropriate sedative drugs need to be given. When the child is relatively stable, the pediatrician injects the contrast agent into the child’s cubital vein, and the radiologist uses the obtained original slices to perform multiplanar reconstruction (MPR), maximum intensity projection (MIP), minimum density projection (Min IP). Thin-layer reconstruction, volume rendering (VR), and other techniques for image post-processing can further detect intracardiac malformations and extracardiac vascular abnormalities. For children who cannot tolerate long-term sedation, C-MRI examination, CCTA combined with echocardiography can also make a systematic judgment for the disease. In addition, the results of CCTA examination are more meaningful in differentiating cardiac tumors and thrombus. CCTA may be considered in children with abnormal coagulation function.

### Fluorodeoxyglucose/PET

In recently years, FDG-PET has been a complementary tool for evaluating patients with cardiac mass and can help differentiate benign from malignant lesions (26). However, this examination is only an optional auxiliary examination, and its clinical application is not common in some areas.

### Plain chest X-ray

This examination can determine whether the cardiac shadow is enlarged and pleural effusion. However, it cannot show the space-occupying space in the cardiac cavity or the space-occupying volume in the myocardial wall, and it is not specific for tumor diagnosis.

### Electrocardiogram

ECG shows no specific changes. However, if the tumor affects the cardiac conduction system, atrial or ventricular premature beats and conduction block may occur, and in severe cases, ventricular tachycardia may occur.

### Cardiac catheterization

Cardiac catheterization is one of the main techniques for detecting heart disease (27). However, it requires invasive operations, may induce complications such as infection and thrombosis and has poor repeatability, which is difficult for infants and young children to tolerate, so it limits its use in infants and young children.

### Pathological biopsy

Though visualization methods have improved continuously, the most vital part of an assessment of prognosis is a histopathological analysis of the tumors, which remains the gold standard. Nevertheless, this approach is limited to patients undergoing surgical resection. There are many differences between the pathological morphology of primary cardiac tumors in the pediatric population and primary cardiac tumors in adult (28).

### Serological examination

There are elevated white blood cell counts and blood platelet counts in laboratory tests. It is common for heart tumors to increase the levels of proinflammatory cytokines (interleukin 6, especially in myxoma) (1). However, these changes were not specific.

## Characteristics of common primary cardiac tumors in children

Each type of tumor has its specificity, and we have made a summary according to the frequency of tumor occurrence (Table 2).

**TABLE 2:** Characteristics, imaging results, and treatment options of different benign cardiac tumors.

Tumors	Preferred age of patient	Preferred gender	Isolated/ Multiple	Predisposing area	Clinical manifestations	Imagine features				Treatment
							
						Echocardiography	C-MRI	CT/CCT	Pathological biopsy	
Rhabdomyoma	<1 year old	No significant sex predilection	Multiple	Wall of the ventricle (atrial) or interventricular septum	(i) Approximately 70–90% of children with CR had a documented tuberous sclerosis; (ii) most children are asymptomatic or have mild symptoms; a small number of patients may experience arrhythmias, heart murmurs, cyanosis, or shortness of breath, and some neonates may experience sudden death.	(i) The local thickening of the myocardium around the tumor leads to the weakening or disappearance of myocardial activity; (ii) tumors can protrude into the cardiac cavity, resulting in varying degrees of changes in cardiac hemodynamics; (iii) the tumor showed moderate intensity, homogeneous, dense echo, regular tumor margins, sessile, and low activity; (iv) easily distinguishable from other types of heart masses	The tumor is isointense on T1-weighted images and hyperintense on T2-weighted images on C-MRI	Cardiac CT usually shows an intramural lesion with homogeneous low attenuation and intraluminal expansion. While CR on enhanced CT of the heart shows that the lesions are low-density areas	(i) Hamartoma composed of cardiomyocytes; (ii) the tumor is round or oval in different sizes; (iii) the boundary with the surrounding tissue is clear, gray-white, and has no capsule; (iv) there are “spider cells”; (v) PAS staining was positive, specific expression of striated muscle characteristic actin, desmin, myoglobin and other immunohisto chemical markers, but not ki-67	Non-surgical treatment: Review echocardio graphy every 1–3 months, and pay attention to whether the tumor has regressed or drug treatment.
Fibroma	<1 year old	Male	Isolated	Ventricle’s interventricular septum and free wall	(i) CF is associated with Gorlin syndrome (also known as neonatal basal cell nevus syndrome); (ii) children may be asymptomatic or experience arrhythmias, heart failure, or sudden death.	CF is usually homogeneous on echocardiography, brighter than the surrounding myocardium; often contain hyperechoic calcifications with a clear border with the surrounding tissue.	Isointense on T1-weighted images and myocardium and low-intensity on T2-weighted images	While cardiac CT showed a solitary, homogeneous soft tissue mass, which could infiltrate to the periphery, and calcification could be seen inside	(i) The specimen is generally round, with a white vortex on the cut surface, similar to uterine leiomyoma, with a clear border, and can grow to infiltrate toward the surrounding tissue.; (ii) microscopic examination	
									shows that lightly stained spindle cells in the cytoplasm grow toward the surrounding myocardial tissue; (iii) there was a lot of collagen matrix around the tumor cells, and the immunohisto chemical markers of the tumor cells were positive for actin and vimentin but negative for SMA, desmin, and S-100.	Surgical treatment: (i) Multiple intraoperative partial excision can be performed to relieve outflow tract obstruction and stenosis to minimize myocardial damage; (ii) heart transplantation may be the only option if the tumor is so large that it invades the ventricle wall extensively and cannot be surgically removed
Myxoma	Often occurs in elder children	Female	Isolated	Left atrium	(i) CM has a familial predisposition, and approximately 10% are associated with Carney syndrome with a high recurrence rate; (ii) children may have clinical symptoms such as dyspnea, palpitation, and pale complexion; (iii) even lung and	(i) Solitary intra-atrial space-occupying lesions, well-defined, pedunculated, lobulated hyperechoic masses, with more significant activity in different heart phases, deformable, and possibly obstructing the mitral valve, Oral or tricuspid valve orifice, possibly with thrombosis	(i) Isointense on T1-weighted images, and increased signal intensity on T2-weighted images and areas of acute hemorrhage appear on both	A well-circumscribed oval intraluminal mass	(i) Consist of scattered cells within a mucopolysac charide matrix originating from a multipotent stroma capable of neural and endothelial differentiation; (ii) the immunohisto chemical markers	Surgical treatment: it should be respected as much as possible.
					cerebral embolism.	and calcification; (ii) occasionally presents as a rare polycystic mass that can easily be misdiagnosed as a hydatid cyst.	T1-weighted and T2-weighted images; (ii) on weighted images, these regions become hyperintense as the hemoglobin is metabolized.		of tumor cells were positive for S-100 and Vimentin; (iii) pedunculated, mobile tumors that are homogeneous and gelatinous, with a smooth, villous, or brittle surface.	
Teratoma	Fetal	Male	Isolated	Commonly in the mediastinum but rarely in the pericardium. Intra-pericardial tumors arise from the base of the heart and are attached to the root of the pulmonary artery and aorta	(i) A progressive decline in cardiac output; (ii) effusions in the pleural and pericardial spaces; (iii) the effusion or mass effect may cause extrinsic compression on the heart and cardiac tamponade	Located inside or outside the pericardium, and the tumor’s diameter is usually 2–9 cm, which protrudes modularly toward the cardiac cavity. The tumor capsule is clear, and cystic-solid is the most common, which is possibly accompanied by severe pericardial effusion	Rarely used	Lipid or calcific densities	The same as teratomas in other parts: mature teratomas are composed of various mature tissues derived from 2 to 3 germ layers.	Surgical treatment: The tumor should be surgically removed as quickly and completely as possible
Hemangioma	No significant age predilection	No significant sex predilection	Isolated	Right atrium	Asymptomatic, myocardial ischemia, stroke, embolization, syncope, arrhythmia, ventricular outflow tract obstruction, or sudden death.	Hypoechoic or anechoic areas inside the tumor, calcification with strong echoes around the tumor, and no abnormal blood flow	(i) High vascularity of CH.; (ii) intermediate and high signal intensity compared with myocardium	Abnormal annular calcification in cardiac shadow	(i) Benign proliferative endothelial cells lining blood vessels with increasing vascularization and intramural infiltration into the surrounding	Due to the diversity of clinical manifestations, the treatment of cardiac hemangioma has not yet reached a unified standard
							in T1- and T2-weighted images.		myocardial walls characterize; (ii) based on the predominant type of proliferating vessels, hemangiomas are classified into cavernous, capillary, and arteriovenous types	

### Cardiac rhabdomyoma

CR occurs almost exclusively in children, mainly before age 1, and can also be found in fetus, with no significant sex predilection (2, 4, 10). In a retrospective analysis by Słowińska et al. (29) CR was detected prenatally in 71/100 cases (71%), and 82/100 cases (82%) were diagnosed within 4 months after birth. Approximately 70–90% of children with CR had a documented tuberous sclerosis (TSC) diagnosis in recent years (29–31), also called Bourneville disease. The discovery of CR can guide the early diagnosis of TSC; in other words, a high level of clinical suspicion of CR should be maintained when a child is diagnosed with TSC. Tumors seen on echocardiography are primarily found in the ventricular wall and interventricular septum, but also the atrial wall and involving valves. The local thickening of the myocardium around the tumor leads to the weakening or disappearance of myocardial activity, and tumors can protrude into the cardiac cavity, resulting in varying degrees of changes in hemodynamics and affecting cardiac systolic and diastolic function. The tumor presents with moderate intensity, homogeneous, and dense echoes, with regular tumor margins, sessile, and low activity; multiple tumors are a prominent feature of CR, and it is not difficult to distinguish them from other types of cardiac mass under ultrasound. It is easier to diagnose CR if there is a history of TSC; Cardiac CT usually shows an intramural lesion with homogeneous low attenuation and intraluminal expansion. While CR on enhanced CT of the heart shows that the lesions are low-density areas, C-MRI isointense on T1-weighted images and hyperintense on T2-weighted images. Histopathologically, it is a hamartoma composed of cardiomyocytes. The tumor body varies in size and is round or oval. It has a clear boundary with the surrounding tissue, is grayish-white, and has no capsule. There are “spider cells” in some areas conducive to disease diagnosis. At the same time, cardiac rhabdoid tumor cells were positive by PAS staining and specifically expressed rhabdoid characteristic actin, desmin, myoglobin, and other immunohistochemical markers but did not express ki-67. Most children with CR have asymptomatic or mild symptoms, and most of them are found during the physical examination; a few patients will have clinical manifestations such as arrhythmia, heart murmur, cyanosis, or shortness of breath, and very few may also experience sudden death, which is more common in neonates.

### Cardiac fibroma

CF is children’s second most common benign cardiac tumor after CR, and is also more common in infants (32, 33). Male has a slight predominance, and usually single tumor. CF is associated with Gorlin syndrome (also known as neonatal basal cell nevus syndrome) (34), an autosomal dominant disorder caused by mutations in the PTCH1 gene. CF is usually homogeneous on echocardiography, brighter than the surrounding myocardium, often contain hyperechoic calcifications, and is usually single tumors with a clear border with the surrounding tissue. The tumors prefer to be located in the ventricle’s interventricular septum and free wall, and fewer occur in the atrium. While cardiac CT showed a solitary, homogeneous soft tissue mass, which could infiltrate to the periphery, and calcification could be seen inside, CF in MRI showed isointense on T1-weighted images and myocardium and low-intensity on T2-weighted images. Markedly enhancing borders and hypointense in the core are characteristic signs of fibroids. The specimen is generally round, with a white vortex on the cut surface, similar to uterine leiomyoma, with a clear border, and can grow to infiltrate toward the surrounding tissue. Microscopic examination shows that lightly stained spindle cells in the cytoplasm grow toward the surrounding myocardial tissue. There was a lot of collagen matrix around the tumor cells, and the immunohistochemical markers of the tumor cells were positive for actin and vimentin but negative for SMA, desmin, and S-100. CF may occupy any heart structure and were primarily seen to grow with a predisposition to the ventricles. The tumor bulk is intramural, occupying the chamber cavity, but its margins were found to interdigitate with ventricular muscles, replacing the functional muscle mass. In this way, they can extend into the ventricular conduction system, interfere with electrical conduction, and cause arrhythmia. Boston Children’s Hospital reviewed 40 years of experience treating primary cardiac tumors and found that ventricular arrhythmias in patients with CF were about 64%, and the recurrence rate of arrhythmias after tumor resection was low (35). Since the base of a CF tumor often infiltrates the free wall of the ventricle and occupies most of the cardiac chambers, it can cause a decrease in the adequate cardiac chamber volume and myocardial contractility, leading to congestive heart failure.

### Cardiac myxoma

CM is another one of the typical primary cardiac tumors in children (36). Dr. Ding’s study (4) reviewed a single-institute 12 years of experience, which showed myxoma often occurs in elder children. CM has a familial predisposition, and approximately 10% are associated with Carney syndrome with a high recurrence rate (37, 38). There is predominance in female, with the incidence ranging from 1.5 to 2 times that in men (39). About 80% of myxomas originate from the left atrium; (1, 40, 41) the rest are primarily located in the right atrium, and the ventricle is rare, accounting for 3–4% and 8% of the left and right ventricles, respectively. Echocardiography is the primary test for diagnosing CM with high accuracy. Ultrasonographic manifestations of CM are solitary intra-atrial space-occupying lesions, well-defined, pedunculated, lobulated hyperechoic masses, with more significant activity in different heart phases, deformable, and possibly obstructing the mitral valve, Oral or tricuspid valve orifice, possibly with thrombosis and calcification. Occasionally presents as a rare polycystic mass that can easily be misdiagnosed as a hydatid cyst; Due to the unique advantages of color ultrasound diagnosis, cardiac CT and cardiac MRI are less used in clinical practice. Contrast-enhanced cardiac CT may present as a well-circumscribed oval intraluminal mass; on MRI, CM is usually isointense on T1-weighted images, and increased signal intensity on T2-weighted images and areas of acute hemorrhage appear on both T1-weighted and T2-weighted images. On weighted images, these regions become hyperintense as the hemoglobin is metabolized. Histologically, tumors consist of scattered cells within a mucopolysaccharide matrix originating from a multipotent stroma capable of neural and endothelial differentiation. The immunohistochemical markers of tumor cells were positive for S-100 and Vimentin. Typical CM is pedunculated, mobile tumors that are homogeneous and gelatinous, with a smooth, villous or brittle surface. The effect of the so-called “wrecking ball” is known. CM often prolapses to varying degrees to the atrioventricular valve opening, causing symptoms and signs of atrioventricular valve stenosis and insufficiency. Children may have clinical symptoms such as dyspnea, palpitation, and pale complexion (1, 3, 41); when heart tumor fragments fall off, lung and cerebral embolism may occur, manifested as limb hemiplegia, impaired speech, etc. Individual children may have acute cerebral embolism as the first presentation (39, 42). Symptoms in some children were abrupt, intermittent, and position-related. Because of the different clinical characteristics of CM, the misdiagnosis rate is high.

### Several other rare primary cardiac tumors

(i) Cardiac Teratoma: Myocardial germ cell tumors are rare, with scattered reports (43). More than half are diagnosed *in utero*, most of the remainder in children under 15 years of age. There is a slight predominance in males. Teratomas are more commonly reported in the mediastinum, but rarely in the pericardium. Intra-pericardial teratomas arise from the base of the heart and are attached to the root of the pulmonary artery and aorta, most of which are mature type (44). Ultrasonographic manifestations show that teratoma can be located inside or outside the pericardium, and the tumor’s diameter is usually 2–9 cm, which protrudes modularly toward the cardiac cavity, The tumor capsule is clear, and cystic-solid is the most common, which is possibly accompanied by severe pericardial effusion; Lipid or calcific densities can also be present on CT, which often provide valuable clues to the diagnosis. The pathological manifestations are the same as teratomas in other parts: mature teratomas are composed of various mature tissues derived from 2 to 3 germ layers. Tumor growth was extraordinarily rapid and associated with a progressive decline in cardiac output (45). Rupture of the cyst in the pericardium leads to the development of effusions in the pleural and pericardial spaces. The effusion or mass effect may cause extrinsic compression on the heart and cardiac tamponade. (ii) Cardiac Hemangioma (CH): CH accounts for 2–3% of the primary cardiac tumors with no significant sex predilection (46). The right atrium is the most common location for fetal and neonatal CH, whereas the left ventricle is the most common site for CH in the adulthood (46, 47). Imaging is helpful in the preoperative screening and diagnosis of CH. Plain chest X-ray and electrocardiography are normal or non-specific in most of cases. Abnormal annular calcification in cardiac shadow is occasionally seen on chest X-ray or cardiac CT. Echocardiography enables real-time observation of CH, which showed that there were hypoechoic or anechoic areas inside the tumor, calcification with strong echoes around the tumor, and no abnormal blood flow. MRI is good at describing the high vascularity of CH. Typically, CH shows intermediate and high signal intensity compared with myocardium in T1- and T2-weighted images. In addition, histopathology is still the gold standard for diagnosing cardiac hemangioma. Histopathologic features of CH are identical to those of hemangiomas elsewhere in the body. Examination shows that benign proliferative endothelial cells lining blood vessels with increasing vascularization and intramural infiltration into the surrounding myocardial walls characterize. Based on the predominant type of proliferating vessels, hemangiomas are classified into cavernous, capillary, and arteriovenous types (48). Hemangioms are histopathologically benign and clinically dangerous tumors with various presentations, including asymptomatic, myocardial ischemia, stroke, embolization, syncope, arrhythmia, ventricular outflow tract obstruction, or sudden death.

## Choice of treatment mode

There is still no unified standard for surgical correction of primary cardiac tumors in infants and young children. Treatment options for pediatric primary cardiac tumors should be performed individually whether children with primary cardiac tumors need surgery and the choice of surgery methods and timing need to be comprehensively dependent mainly on the size, location, nature of the tumor, and its impact on hemodynamics. In the case of benign tumors, the most commonly reported indication for surgical intervention was “clinical symptomatology,” which usually representing severe obstruction with hemodynamic compromise or arrhythmias, such as significant blood flow disorder, hypotension, the need for inotropic drugs, or even unable to escape ECMO treatment.

### Non-surgical treatment

When the cardiac tumor is small in size, slow in growth, and does not affect the vital signs of the child, regular follow-up can be performed, and echocardiography can be reviewed in 1–3 months to evaluate the cardiac function of the child and monitor the changes of the tumor. For example, CR cells have no mitotic ability, so some cases can spontaneously regress with the prolongation of the disease course (49, 50), and drug therapy (such as Rapamycin) may reduce the tumor, so the current treatment for CR is symptomatic drug therapy. If there is no continuous remission, consider complete or partial tumor resection by surgery.

### Surgical treatment and surgical approach

Surgery is the traditional treatment method, but the indications for surgery need to be carefully grasped. The principle purpose of surgical treatment is to restore normal hemodynamics and protect important structures and cardiac tissue. When surgical intervention is deemed necessary, the appropriate surgical approach selection depends on the tumor’s location. It aims for an adequate exposure that would permit a complete resection with adequate margins and minimal disruption of healthy cardiac tissue and anatomy. It is worth noting that compared with adults, infants and young children have smaller and thinner heart walls, increased load after tumor resection, decreased contractility, and are more prone to hemodynamic complications. Therefore, the surgical plan needs to be individually formulated (4, 51); Gentle manipulation of the heart is required during the procedure to prevent the mass from breaking up and embolizing. Tumors should be resected through the natural channel whenever possible, avoiding ventricular incision, especially the left ventricle. This minimizes myocardial damage, avoids damage to coronary arteries, valves, and conduction systems. In recent years, thoracoscopy and small-incision cardiac surgery have gradually matured, significantly reducing the trauma of children. Patients with large tumors may require heart transplantation (52).

For instance, CF is generally not likely to regress spontaneously (51) and require a limited period of surgical treatment; the base of CF is often broad, and the boundary with the myocardial tissue is unclear. Multiple intraoperative partial excision can be performed to relieve outflow tract obstruction and stenosis to minimize myocardial damage. Heart transplantation may be the only option if the tumor is so large that it invades the ventricle wall extensively and cannot be surgically removed (53); CM is associated with a high risk of embolism and is prone to recurrence, so it should be respected as much as possible. Most CMs are pedunculated, have clear borders, and are easy to distinguish from normal myocardial tissue, and most of them can be wholly resected (54); teratoma proliferates and has apparent compression to the heart. Once found, it should be surgically removed as soon as possible (45), and some cases were reported underwent open fetal surgery. At the same time, complete surgical resection is the preferred treatment. Chemotherapy and radiotherapy are not very useful in teratoma. Due to the diversity of clinical manifestations, the treatment of cardiac hemangioma has not yet reached a unified standard; accurate localization of the tumor location before surgery is crucial (47). Surgical treatment of atrial hemangioma is the best, the recurrence rate is low, and the patient’s symptoms are significantly improved or completely relieved (55).

#### Surgical results

Good results may be obtained after complete resection of early benign cardiac tumors with a low recurrence rate. Postoperative complications are mainly cardiac insufficiency (low cardiac output), infection, and damage to adjacent structures or nerves. Although surgical treatment carries a high risk of death and postoperative complications in the perioperative period, most patients eventually survive. The patient survived well at postoperative follow-up. Death and postoperative complications tend to appear in younger children (23). Therefore, when conditions permit, younger children should be treated conservatively and followed close until there is sufficient cardiac reserve to tolerate surgery.

#### Precautions after operation

However, most tumors cannot be completely removed (10, 46, 51). For patients with incomplete resection, dynamic echocardiography is required to monitor whether there is recurrence or whether the residual tumor volume continues to grow, hemodynamic changes, and myocardial beating. If there is a transient low output state of low cardiac output, continuous blood purification (CBP) or extracorporeal membrane oxygenation (ECMO) can be applied to improve perfusion.

## Outcomes and follow-up

A higher percentage of tumor relapse was noted for myxoma and teratoma (4, 8). In recent years, literature reports have shown that with the advancement of medical technology, the detection rate and successful treatment rate of cardiac tumors have increased year by year, and surgical techniques have been improved. The patients’ quality of life has improved.

## Conclusion

Most primary cardiac tumors in children are benign; most were diagnosed in infancy, with various clinical manifestations: dyspnea and chest pain are reported among the most common symptoms. There are many types of tumors, all of which have specific typical imaging and pathological features. Although echocardiography has provided a consistent assessment of anatomy and function, CT and MRI currently allow more extensive diagnoses. Diagnosing a heart tumor in children is not synonymous with a fatal prognosis; complete surgical resection is the most valuable treatment. Ensuring cardiac function during surgery and long-term follow-up has a good prognosis.

## Author contributions

CQ-S conceptualized the study, drafted the initial manuscript, and reviewed and revised the manuscript. CF-Y and YK-C collected literatures. YM-L critically reviewed the manuscript. All authors have read and approved the final manuscript as submitted, agreed to be accountable for all aspects of the work in ensuring that questions related to the accuracy or integrity of any part of the work are appropriately investing and resolved.

## Abbreviations

CBP: Continuous blood purification
CCT: Chest computed tomography
CCTA: Cardiac CT angiography
CEUS: Contrast-enhanced myocardial ultrasonography
CF: Cardiac Fibromas
CH: Cardiac Hemangioma
CM: Cardiac Myxomas
C-MRI: Cardiac magnetic resonance imaging
CR: Cardiac Rhabdomyoma
ECG: Electrocardiogram
ECMO: Extracorporeal membrane oxygenation
FDG-PET: Fluorodeoxyglucose/PET
MIP: Maximum intensity projection
Min IP: Minimum density projection
MPR: Multi-plane reconstruction
TEE: Transesophageal echocardiography
TTE: Transthoracic echocardiography
TSC: Tuberous sclerosis
UCG: Ultrasonic cardiogram
VR: Volume rendering
WHO: World Health Organization.
